# Inflammatory Response Against *Staphylococcus aureus via* Intracellular Sensing of Nucleic Acids in Keratinocytes

**DOI:** 10.3389/fimmu.2022.828626

**Published:** 2022-02-24

**Authors:** Quang Vinh Ngo, Larissa Faass, Aline Sähr, Dagmar Hildebrand, Tatjana Eigenbrod, Klaus Heeg, Dennis Nurjadi

**Affiliations:** ^1^ Department of Infectious Diseases, Medical Microbiology and Hygiene, Heidelberg University Hospital, Heidelberg, Germany; ^2^ Deutsches Zentrum für Infektionsforschung (DZIF), Department of Infectious Diseases, Heidelberg University Hospital, Heidelberg, Germany; ^3^ Max von Pettenkofer Institute, Chair for Medical Microbiology and Hygiene, Ludwig Maximilians University Munich, Munich, Germany

**Keywords:** *Staphylococcus aureus*, *Staphylococcus epidermidis*, keratinocyte, skin immune response, bacterial RNA, host-pathogen interaction

## Abstract

*Staphylococcus aureus* is one of the clinically most relevant pathogens causing infections. Humans are often exposed to *S. aureus*. In approximately one-third of the healthy population it can be found on the skin either for long or short periods as colonizing “commensals”, without inducing infections or an inflammatory immune response. While tolerating *S. aureus* seems to be limited to certain individuals and time periods in most cases, *Staphylococcus epidermidis* is tolerated permanently on the skin of almost all individuals without activating overwhelming skin inflammation. To investigate this, we co-cultured a keratinocyte cell line (HaCaT) with viable *S. aureus* or *S. epidermidis* to study the differences in the immune activation*. S. aureus* activated keratinocytes depicted by a profound IL-6 and IL-8 response, whereas *S. epidermidis* did not. Our data indicate that internalization of *S. aureus* and the subsequent intracellular sensing of bacterial nucleic acid may be essential for initiating inflammatory response in keratinocytes. Internalized dsRNA activates IL-6 and IL-8 release, but not TNF-α or IFNs by human keratinocytes. This is a non-specific effect of dsRNA, which can be induced using Poly(I:C), as well as RNA from *S. aureus* and *S. epidermidis*. However, only viable *S. aureus* were able to induce this response as these bacteria and not *S. epidermidis* were actively internalized by HaCaT. The stimulatory effect of *S. aureus* seems to be independent of the TLR3, -7 and -8 pathways.

## Introduction

The immunology of the human skin is complex. The skin is the largest immune organ of the human body and acts as a physical and immunological barrier against invading pathogens, which can sense invading pathogens and react accordingly (1, 2). As a primary response and innate defense mechanism, keratinocytes respond to stimuli, such as trauma and bacterial invasion, by producing antimicrobial peptides (AMP) to eliminate potentially harmful pathogens.

Although the human skin is constantly exposed to various bacteria as part of the skin microbiome or invading pathogen, some bacteria are tolerated, while others are eliminated. For approximately 20-30% of the healthy population, *Staphylococcus aureus* is considered part of their microbiome and colonize their nasal cavity and the skin (3). At the same time, others are transiently colonized and can prevent persistent colonization with *S. aureus* (4).

The underlying mechanism of *S. aureus* colonization is complex and multifactorial. Potential determinants include bacterial virulence factors (5), the composition of and competition within the nasal microbiome (6, 7) and immunological processes involving the skin and the adaptive immunity (6, 8–13). However, the link between the innate and the adaptive immune response in this context is not yet fully elucidated. The skin is equipped with an arsenal of pattern recognition receptors (PRRs), which sense conserved microbial structures to initiate innate immune response, and produce pro-inflammatory cytokines and chemokines, which is essential for leukocyte recruitment and activation of adaptive immune response. Although *Staphylococcus epidermidis*, commonly regarded as commensals and *S. aureus* as facultative pathogens share similar conserved microbial structures, only about one-third of the healthy population is colonized by *S. aureus*, whereas *S. epidermidis* can be found in nearly all individuals (14, 15). This observation leads us to the hypothesis that *S. aureus* and *S. epidermidis* induce a differential immune response in the skin, facilitating or preventing persistent colonization.

Classically, the recognition of staphylococci by innate immune cells, such as keratinocytes, is attributed to secreted proteins (e.g. protein A, hemolysins, phenol-soluble modulin (PSM)) and cell wall components (e.g. peptidoglycan and lipoteichoic acid) *via* the extracellular membrane-bound PRR toll-like receptor 2 (TLR2) (16–19). In *S. aureus*, TLR2 activation has been demonstrated to depend on PSM-mediated shedding of lipoproteins (20). Of note, species, and strain differences in PSM expression due to chromosomally or extra-chromosomally encoded PSM may have an influence on immune evasion and activation (21). However, since both *S. aureus* and *S. epidermidis* can activate the extracellular TLR2 to initiate the skin’s immune response against bacterial invasion and infection (16, 19), another mechanisms must be involved in differentiation between *S. aureus* and *S. epidermidis*. For a long time, *S. aureus* has been regarded as an extracellular pathogen. The significance of *S. aureus* as an intracellular pathogen has only been acknowledged recently (22, 23). Indeed, *S. aureus* can survive and persist within keratinocytes (24–26) and the intracellular presence may have some importance in the pathophysiology of colonization and infection (24). The activation of the intracellular PRR, nucleotide-binding oligomerization domain-containing protein 2 (NOD2) by *S. aureus* has been demonstrated to be involved in the induction of antimicrobial peptides in keratinocytes (27) and may play a role as a microbial sensor in discriminating between commensal and pathogenic bacteria (28). In contrast, *S. epidermidis* is generally regarded as a non-invasive facultative pathogen, mainly causing superficial and device-associated infections (24).

This study aims to investigate, how keratinocyte can sense and differentiate between commensal and potentially pathogenic staphylococci in an *in vitro* experimental set up using immortalized keratinocytes.

## Materials and Methods

### Keratinocyte Cell Culture

Immortalized keratinocyte cell line HaCaT (DKFZ, Germany) was sustained in proliferative state in a low Ca^2+^ (0.036mM Ca^2+^) DMEM (Invitrogen) supplemented with 10% fetal bovine serum and 5 mM L-glutamine without antibiotics at 37°C with 5% CO_2_.

### Stimuli and Receptor Ligands

For the *in vitro* infection assays, 1x10^5^ or 1x10^6^ HaCaT cells were seeded into a 24-well plate or a 12-well plate, respectively, and cultured to confluence at 37°C with 5% CO_2_ for 24 hours. The following stimulating agents were used: lipopolysaccharide (LPS) 0.5 µg/ml (Invivogen), Pam3Cys (P3C) 0.5 µg/ml (EMC microcollections GmbH), resiquimod (R848) 5 µg/ml (Enzo Life Sciences GmbH), CpG oligodeoxynucleotides (CpG) 1 µM (MWG-Biotech AG), polycytidylic acid (Poly(I:C)) 1 μg/ml (Invivogen), peptidoglycan (PGN) 10 μg/ml (Invivogen) and muramyldipeptide (MDP) 5 μg/ml.

### *S. aureus* Preparations for Infection Experiments

Bacterial cultures were maintained in either tryptic soy broth (TSB) (Becton Dickinson) as a liquid culture or on Columbia blood agar plate (supplemented with 5% sheep’s blood). Invasive *S. aureus* strains USA300 FRP3757, 8325-4 and its isogenic non-invasive fibronectin-binding protein A and B (FnBA/B)-deficient mutant (Δ*fnbA*Δ*fnbB* 8325-4) DU5883, and *S. epidermidis* (ATCC^®^35894) were used in the initial experiments of this study. Verification experiments were conducted using additional clinical bacterial isolates; an invasive clinical *Staphylococcus argenteus* SA147 (a member of the *S. aureus* complex), an invasive clinical *S. aureus* SS-11921, a non-invasive clinical *S. aureus* isolate SA303, and a non-invasive coagulase-negative *Staphylococcus lugdunensis* HD1. A summary table for all used strains in this study is provided in the **Supplementary Table S1**. For the infection assay with living staphylococci, bacterial cells cultured in tryptic soy broth were harvested in the mid-log phase, adjusted to a McFarland 3.0 (equivalent of 9x10^8^ CFU/ml) and diluted to the desired concentrations. After 2 hours of initial incubation with keratinocytes at 37° with 5% CO_2_, gentamicin and lysostaphin (Genaxxon GmbH, Germany) were added to the co-culture to an end-concentration of 10µg/ml and 2.5µg/ml, respectively, to ensure optimal removal of extracellular bacteria. After further 22 hours of incubation (for a total of 24 hours of co-culture), the cell supernatant was removed and stored at -20°C until further use. The remaining cells were washed thrice with sterile PBS, resuspended in cell lysis buffer (RNA Mini Kit, Bio&Sell GmbH, Germany) and stored at -80°C until further use.

For infection experiments with heat-killed bacteria, *S. aureus* or *S. epidermidis* were heat-inactivated at 95°C for 60 min. Viability check was performed by plating of 10µl of inactivated bacterial suspension onto Columbia blood agar followed by 24 hours incubation at 37°C. *S. aureus* conditioned medium was generated by cultivation of *S. aureus* in DMEM with 10%FCS without antibiotics supplement for 24 hours under constant agitation (200rpm). After centrifugation at 3000 rpm for 10 minutes, the supernatant was sterile filtered (Millex^®^-GS Filter; 0.22 μm). 50% v/v of bacterial supernatant was used as stimulant. To assess the toxicity of 50% v/v bacterial supernatant, we performed an MTT assay on a titration experiment with 10%, 20%, 30%, 40% and 50% v/v bacterial supernatant. Briefly, 5x10^4^ HaCaT cells (per well) were seeded into 48 well plate and cultured for 1 day at 37°C and 5% CO_2_. After reaching a confluency of 75%, cells were incubated with different amounts of bacterial supernatants (10-50%) and was incubated further for another 24 hours. Then, supernatants were removed and CellTiter 96^®^ Aqueous One Solution Cell Proliferation Assay Solution (Promega; Walldorf, Germany) was added according to the manufacturer’s instructions. After 1 hour of incubation absorbance was measured at 490nm. Cell viability was normalized to an unstimulated control, expressed as percent (**Supplementary Appendix**).

### Quantification of Intracellular Bacteria

To quantify internalized bacteria by flow cytometry, bacterial cells were fluorescently stained using carboxyfluorescein succinimidyl ester (CFSE, ThermoFisher Scientific, Germany). Briefly, bacterial cells harvested in the mid-log phase were suspended in PBS with 2µM CFSE were incubated in the dark at 37°C under constant agitation of 200 rpm for 20 minutes, followed by several PBS washing steps (3 times). The infection experiment was performed as described above. For quantification, infected keratinocytes with CFSE-labelled bacteria were trypsinized for 10 minutes and resuspended in PBS. Quantification of internalized CFSE-labelled bacterial cells was performed by flow cytometry with a FACS Canto (Becton Dickinson GmbH, Germany) and with the BD FACSDiva software version 8.0.1 (Becton Dickinson GmbH). First, a gate was set on living cells (excluding cell debris) in the FSC/SSC display. Then, the gated cells were analyzed for FITC-positivity (y-axis: FITC-signal, x-axis: FSC) using the non-infected control as a reference. FITC signal above the defined gate was considered positive. Cell viability was quantified using Annexin V and propidium iodide staining (ThermoFisher Scientific, Germany). Internalization efficiency was expressed as percent of infected cells (CFSE+) over the gated population in the FITC-forward scatter plot (**Supplementary Figure S1**). The invasive *S. aureus* Cowan I and the non-invasive *Staphylococcus carnosus* TM300 were used as positive and negative control for the flow cytometric analysis, respectively. In parallel, manual colony counting to quantify the bacterial density was performed by serial dilution of cell lysate (with 1%Triton X-100) in sterile 0.9% NaCl and plating of 100µl lysate on Columbia blood agar. Bacterial density was manually counted after 24 hours of incubation at 37°C and was expressed as colony forming unit per ml (CFU/ml). Inhibition of bacterial internalization was performed by the addition of 0.5 µM cytochalasin-D one hour prior to infection. The toxicity of 0.5 µM cytochalasin-D was determined in a titration assay (**Supplementary Figure S2**).

### Bacterial RNA Preparation for Stimulation

RNA from bacteria cultured in tryptic soy broth (37°C ambient air, constant shaking 200 rpm) harvested in mid-log growth phase was isolated using the phenol-chloroform extraction method. Briefly, bacterial suspension was homogenized using 0.1mm glass beads in a bead beater (2x30s) after prior washing and resuspension in Tris-EDTA Buffer. After 30 min of shock frosting at -80°C, phenol and chloroform (5:1) were added. The aqueous phase was removed and purified with a column-based RNeasy Kit (Qiagen GmbH, Germany) according to manufacturer’s protocol. DNase treatment was performed by DNase I (Roche GmbH, Germany). Finally, RNA quantity and purity were evaluated in Nano Drop 1000 3.8.1 (ThermoFisher scientific). For transfection, RNA was complexed with DOTAP (1µg RNA with 6µl DOTAP, 3µg and 6µg RNA with 12µl DOTAP) in Opti-MEM for 10-15 min at room temperature prior to stabilization with Ca^2+^ free DMEM without antibiotics. To verify the specificity of RNA stimulation, RNA was inactivated by heat (30 min at 95°C) and RNase A (Promega, Germany) treatment prior to transfection. As an additional control, RNA was inactivated by RNase A 2 hours post-transfection to verify internalization of bacterial RNA.

### Quantitative mRNA Cytokine Expression

To quantify the mRNA expression of genes of interest in HaCaT cells, RNA was extracted from harvested cells using RNA Mini Kit according to the manufacturer’s protocol (Bio&Sell GmbH, Germany) and transcribed to cDNA (First Strand cDNA Synthesis Kit, Thermo Fisher; USA). RTq*-*PCR was performed in duplicates using SYBR Green (Nippon Genetics; Germany) on the Step One Plus machine according to the manufacturer’s protocol and standard cycling conditions (annealing temperature of 60°C). Primers used in this study are listed in **Supplementary Table S2**. For keratinocytes, β-actin served as housekeeping gene.

### Quantification of Cytokines in Cell Supernatant by ELISA

Secretion of various cytokines (IL-6, IL-8, TNF-α, IFN-α and IL-1β) in supernatant from the infection experiments of HaCaT cells were quantified by ELISA in duplicates (BD OptEIA, Becton Dickinson, Germany) according to the manufacturer’s instructions. ELISA were performed on supernatants, which has been frozen at -20°C.

### Western Blot

After infection, keratinocytes were lysed in RIPA buffer. Equal amounts of the lysates were fractioned by SDS-PAGE and electro-transferred to nitrocellulose membranes (neoLab;Heidelberg, Germany). The membranes were blocked with BlueBlock (Serva; Heidelberg, Germany) and incubated with antibodies against RIG-I and MDA-5 (1:1000 overnight; Cell Signaling; Frankfurt am Main, Germany). Detection was visualized by enhanced chemiluminescence (ECL; 7Bioscience; Neuenburg am Rhein, Germany) using a ChemoStar Plus Imager (Intas; Göttingen, Germany).

### Quantification of Protein Bands in Western Blots

8-bit JPEG images were black/white inverted, and bands were quantified *via* ImageJ software version 1.530. In ImageJ the image was adjusted to a background of 0. Then integrated density (IntDen) of each band of interest was quantified by using equal areas for gating. Data were analyzed by GraphPad Prism v9 (USA). The IntDen of protein band of interest was divided through IntDen of the respective houskeeping protein band. For calculation of induction mean of data of three unstimulated samples were set to 100 percentage.

### Statistical Analysis

Visualization of experiment results and statistical analysis were conducted with GraphPad Prism (version 9.0, GraphPad Software Inc, USA). Group comparisons and statistical significance of differences between observations were assessed by two-way-ANOVA or Wilcoxon rank sum test for nonparametric variables as deemed suitable. All experiments were performed in biological triplicates and technical duplicates for each biological replicate. Data presented are summaries of at least three biological replicates, performed as independent experiments. P-values of <0.05 were considered statistically significant.

## Results

### Differential IL-6 and IL-8 Response by *Staphylococcus aureus* and *Staphylococcus epidermidis* in Keratinocytes

To investigate whether keratinocytes react differently to *S. aureus* and *S. epidermidis*, we co-cultured keratinocytes with *S. aureus* (USA300) or *S. epidermidis* (MOI 10 or MOI 100). The next day supernatant was analyzed for secreted inflammation factors.

**Figure 1:**
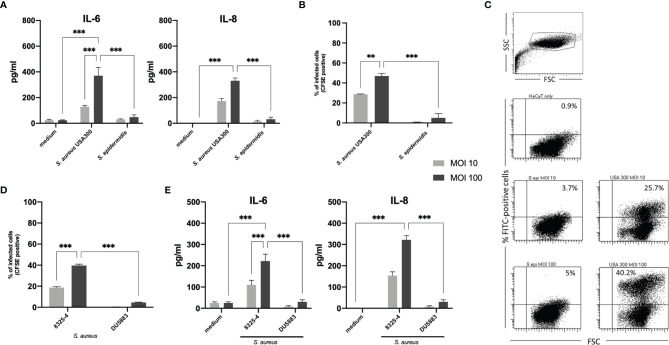
IL-6 and IL-8 response in keratinocytes stimulated with viable *Staphylococcus aureus* and *Staphylococcus epidermidis*. **(A)** *S. aureus* induced significantly more IL-6 and IL-8 than *S. epidermidis* in a dose-dependent manner, which correlated with the amount of internalized bacterial cells following 24 hours exposition in a gentamicin/lysostaphin protection assay. **(B)** Internalized bacteria were quantified by flow cytometry (CFSE positivity). **(C)** Representative experiment of flow cytometry quantification of bacterial internalization. **(D)** To confirm the importance of internalized bacterial cells in inducing IL-6 and IL-8 in our experimental setting, we performed the infection assay with an invasive *S. aureus* strain 8325-4 and the non-invasive isogenic strain *S. aureus* DU5883 with deletions in the fibronectin binding protein genes *fnbA* and *fnbB* (Δ*fnbAB* 8325-4). **(E)** The deletion mutant induced significantly lower IL-6 and IL-8 compared to the wild type. For panels **(A, B**, **D, E)**, grey bars indicate MOI 10 and black bars indicate MOI 100. All experiments were performed as independent experiments in biological triplicates, each in technical duplicates. Statistical significance was calculated using two-way ANOVA. Only statistically significant differences (**p ≤ 0.01, ***p ≤ 0.001) are displayed.

IL-6 and IL-8 were the most prominent and significantly upregulated cytokines detected by ELISA in the 24-hour supernatant (2-hour co-culture with viable bacteria and another 22-hour after addition of gentamicin/lysostaphin) following stimulation with viable *S. aureus* (USA300) but were not detected after stimulation with *S. epidermidis* (**Figure 1A**) IL-1β and TNF-α were not detected in the supernatants (data not shown). There were no significant differences in the induction of antimicrobial peptides of the skin, human beta-defensins (hBD-) 1 to 3, between *S. epidermidis* and *S. aureus* (**Supplementary Figure S3**). In *S. aureus*, the secreted IL-6 and IL-8 levels were dose-dependent and were more profound in co-culture experiments with MOI 100 than MOI 10. There were no significant differences in the proportion of viable cells (Annexin V/Propidium Iodide staining) after stimulation with USA300 and *S. epidermidis* (**Supplementary Appendix **and **Supplementary Figure S4**).

### Internalization of Bacterial Cells Correlates With IL-6 and IL-8 Response

Having confirmed the differential activating ability of USA300 *S. aureus* (FPR3757) and *S. epidermidis* (ATCC^®^ 35894) used in this study, we then investigated possible reasons for this observation. Both staphylococci can activate the extracellular TLR2 to initiate the skin’s immune response against bacterial invasion and infection (16, 19). However, while *S. aureus* can survive and persist within keratinocytes (24–26), *S. epidermidis* is generally regarded as a non-invasive facultative pathogen (24). Therefore, we next investigated whether the intracellular localization of *S. aureus* and *S. epidermidis* may explain the observed differential IL-6 and IL-8 response.

For this purpose, we analyzed internalized bacteria using fluorescent (CFSE)-labelled staphylococci by flow cytometry and manual colony counting. After co-culture with CFSE-labelled *S. aureus* (USA300) or *S. epidermidis* (MOI 10 or MOI 100) for 2 hours, gentamicin and lysostaphin was added and washed multiple times with PBS to kill and remove extracellular bacteria. CFSE signal of internalized bacteria was quantified by flow cytometry (**Figures 1B, C**). A representative dot blot in **Figure 1C** as well as the associated quantification shows that a large proportion of keratinocytes internalized *S. aureus* (mean MOI 10 = 28.8% and MOI 100 = 47.0%), whereas significantly less cells (mean MOI 10 = 0.8% and MOI 100 = 5.1%) contained *S. epidermidis.* Thus, the internalization of bacteria correlated with induced cytokine release (**Figure 1A**). The results of the manual colony counting are summarized in the **Supplementary Appendix** and **Supplementary Figure S5**.

To confirm this correlation, we repeated the experiments using invasive and non-invasive isogenic strains of *S. aureus* 8325-4 and DU5883. *S. aureus* DU5883 is a fibronectin-binding protein A and B deficient mutant of *S. aureus* 8325-4 (8325-4Δ*fnbAB*), which has been shown to have reduced invasive properties (29). **Supplementary Figure S1** and **Figure 1D** confirms that non-invasive DU5883 was only marginally detected in the high MOI of 100 inside of cells. Invasive 8325-4 could be detected in around 20% at MOI 10 and around 40% at MOI 100. Quantification of IL-6 and IL-8 by ELISA showed indeed that invasive *S. aureus* 8325-4 induced IL-6 and IL-8, whereas the non-invasive Δ*fnbAB* mutant did not (**Figure 1E**), thus confirming the correlation between invasion and keratinocyte activation.

**Figure 2:**
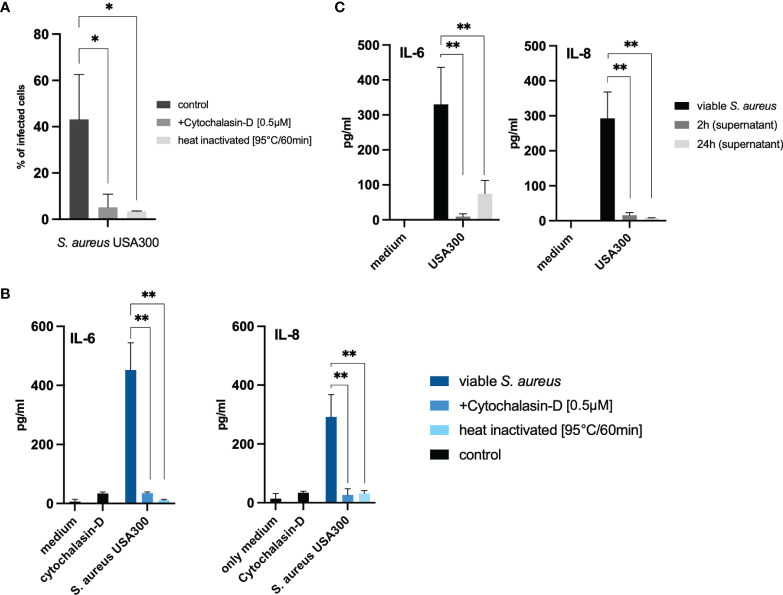
Internalized bacteria and cellular contact are necessary to induce IL-6 and IL-8 in keratinocytes. **(A)** Inhibition of bacterial cell internalization (MOI 100) using cytochalasin-D as well as heat inactivation of bacterial cells reduced the number of internalized bacteria and **(B)** diminished both IL-6 and IL-8 response in our experimental set-up. **(C)** Stimulation using 50% (v/v) bacterial supernatant, acquired by culturing bacteria in liquid culture for 2 and 24 hours, did not induce IL-6 or IL-8 significantly. All experiments were performed as independent experiments in biological triplicates, each in technical duplicates. Statistical significance was calculated using Wilcoxon rank sum test. Only statistically significant differences (*p < 0.5, **p ≤ 0.01) are displayed.

Furthermore, inhibition of bacterial internalization by heat inactivation and inhibition of the actin polymerization, that is needed for bacterial invasion (30), using Cytochalasin-D significantly reduced cytokine release into the co-culture (**Figure 2A**). We also excluded that secreted bacterial components are responsible for keratinocyte activation and stimulated cells with *S. aureus* supernatant. In our experimental set-up, 50% v/v of bacterial supernatant was used. The highest non-toxic concentration of supernatant (50% v/v) was chosen after titration and MTT cell viability assay (31) to closely mimic the abundance of proteins and cellular products in the viable bacteria co-culture set-up (**Supplementary Figure S6**). The subsequent experiment showed that stimulation with bacterial conditioned medium (BCM) did not induce IL-6 nor IL-8 response, indicating that cellular contact is essential (**Figures 2B, C**).

### Pro-Inflammatory Response of Keratinocytes to Invading Bacteria Is RNA-Mediated and Species Non-Specific

Since the internalization of bacterial cells was a prerequisite for the induction of the IL-6 and IL-8 response in our experimental set-up, we then investigated whether the activation of intracellular pathogen-sensing receptors might be involved in initiating immune response towards invasive staphylococci.

**Figure 3:**
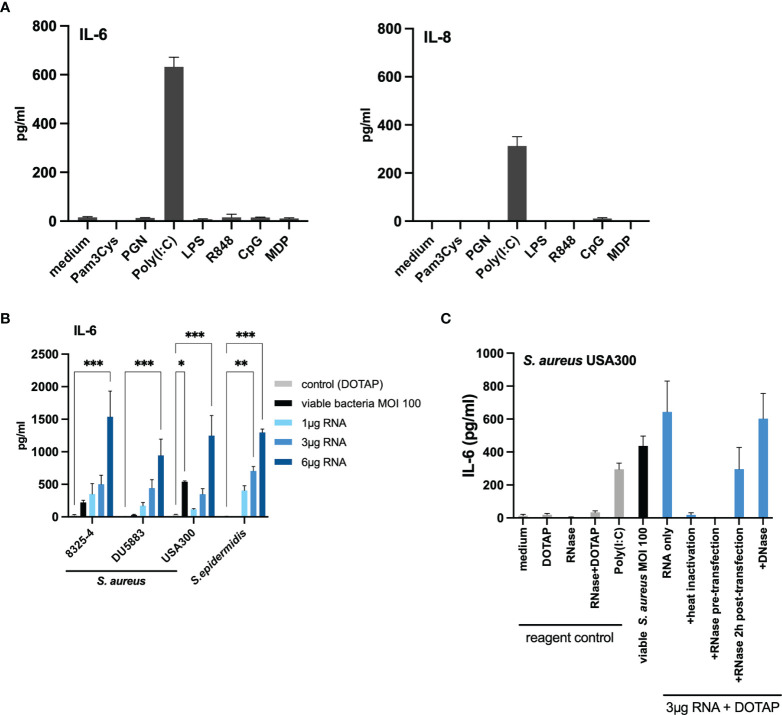
Transfection of bacterial RNA from non-invasive staphylococci induced IL-6 in a similar manner to invasive living bacteria. **(A)** Stimulation with various pattern recognition receptor ligands indicated that TLR3/dsRNA induces IL-6 and IL-8 response in HaCaT keratinocytes. **(B)** Transfection of bacterial RNA isolated from the non-invasive *S. aureus* DU5883 and *S. epidermidis* induced IL-6 in a dose-dependent manner similar to invasive S. aureus strains (USA300, 8325-4). **(C)** RNA inactivation (heat and RNase treatment) prior to transfection diminished the IL-6 response, while RNase treatment 2 hours post transfection did not. There was no effect on the IL-6 induction following DNase treatment suggesting that internalized bacterial RNA is responsible for the IL-6 response. Data for IL-8 are available in the **Supplementary Appendix**. All experiments were performed as independent experiments in biological triplicates, each in technical duplicates. Statistical significance was calculated using two-way ANOVA. Statistically significant differences are indicated by * (*p < 0.5, **p ≤ 0.01, ***p ≤ 0.001).

Host eukaryotic cells are able to sense danger signals *via* various intracellular receptors. In the endosome, nucleic acid (RNA) can be recognized by TLR3, TLR7 and TLR8, while TLR9 recognized the unmethylated cytidine-phosphate-guanosine (CpG) motifs in bacterial DNA (32, 33). TLR3 senses double-stranded RNA (dsRNA) (34), whereas TLR7 and TLR8 sense degradation products of single-stranded RNA (35). In the cytosol, the key sensors for RNA recognition are RIG-I and MDA5, two helicases of the DExD/H motif family, which sense dsRNA (36). Therefore, we next investigated whether various PRR ligands can activate keratinocytes and mimic the initial findings of the co-culture with viable bacteria. We stimulated HaCaT cells using various PRRs ligands, namely Pam3Cys for TLR1/2, Poly(I:C) for TLR3, LPS for TLR4, R848 for TLR7/8, CpG for TLR9 and a nucleotide-binding oligomerization domain-containing protein 2 (NOD-2) ligand. Interestingly, only the TLR3-activator Poly(I:C) induced a significant IL-6 and IL-8 response (**Figure 3A**). This led us to the hypothesis that the observed *S.aureus*-stimulated activation of keratinocytes may be mediated by bacterial RNA in the intracellular compartment.

To verify this hypothesis, we isolated total RNA from the invasive *S. aureus* USA300 and the non-invasive/non-internalized *S. epidermidis* in the logarithmic growth phase and performed titration experiments by transfecting 1µg, 3µg and 6µg of the bacterial RNA using the liposomal transfection reagent DOTAP. Indeed, we observed a dose-dependent IL-6 and IL-8 response (**Figure 3B** and **Supplementary Figure S7**) for both *S. aureus* USA300 and *S. epidermidis*. Of note, although the co-culture with *S. epidermidis* at MOI 100 did not induce IL-6 response, the transfected RNA was able to induce significant IL-6 and IL-8 response. For the invasive *S. aureus* USA300, 3µg RNA could induce a similar IL-6 response as infection with viable bacteria at MOI 100.

To verify if the response was RNA-mediated and was not due to other structures (proteins, polysaccharides, and other cellular structures), we performed RNA inactivation experiments with the RNA of *S. aureus* USA300 (**Figure 3C**). Degradation of bacterial RNA by heat (95°C for 30 mins) and RNase treatment (60°C for 1 hour) prior to transfection abolished the IL-6 response, whereas DNase treatment had no significant effect (IL-6 response was not abolished), which suggests that DNA sensing was not responsible for the induction of pro-inflammatory response following bacterial internalization by keratinocytes. Furthermore, RNase treatment 2 hours post-transfection did not abolish the IL-6 response, thus confirming that internalized bacterial RNA and not extracellular RNA was responsible for the observed IL-6 response (**Figure 3C**). We verified this finding with the isogenic *S. aureus* isolates 8325-4 and its Δ*fnbAB* mutant, DU5883. Similar to *S. epidermidis*, DU5883 did not demonstrate remarkable induction of IL-6 in HaCaT cells, but the experimentally transfected RNA induced similar IL-6 response as the invasive isogenic counterpart (8325-4) (**Figure 3B**).

Similar results were obtained for IL-8 (**Supplementary Figure S7**). Thus, the results of our experiments so far suggest that bacterial RNA in the intracellular compartment may be responsible for inducing pro-inflammatory response in invasive staphylococci.

### Verification of Findings Using Other Invasive and Non-Invasive Clinical Staphylococci

**Figure 4:**
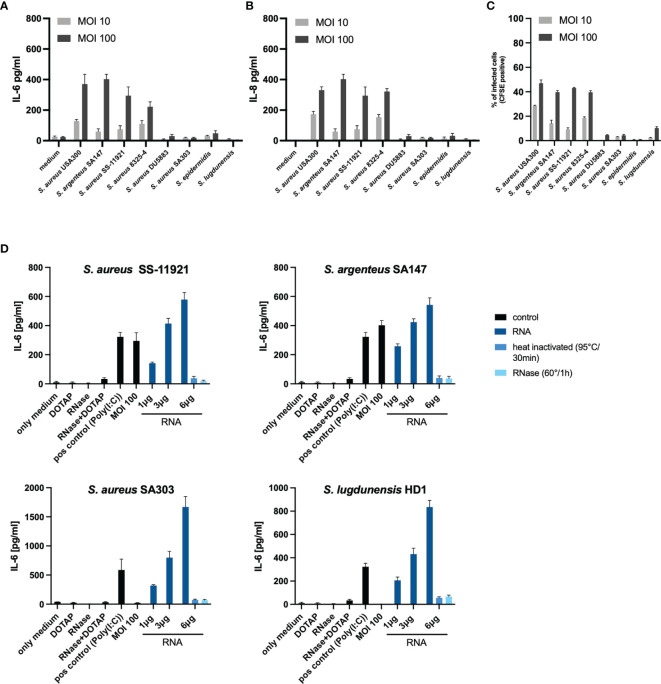
Validation of IL-6 and IL-8 response due to internalized bacterial RNA using clinical staphylococci isolates. To validate our initial findings, we repeated the experiments using four clinical staphylococci isolates; an invasive *S. aureus* clinical isolate SS-11921, an invasive *Staphylococcus argenteus* SA147 (member of the *Staphylococcus aureus* complex), a non-invasive clinical *S. aureus* isolate SA303 and a non-invasive *Staphylococcus lugdunensis* HD1 (coagulase-negative *Staphylococcus*). **(A)** overview of the IL-6 response and **(B)** IL-8 response in a co-culture set-up at MOI 10 and MOI 100, including the four isolates used in the initial experiments. **(C)** Summary of the invasion property (assessed using flow cytometry) quantified as percentage of infected cells (CFSE positive over the gated population). **(D)** Stimulation with viable bacteria for *S. aureus* SA303 and *S. lugdunensis* HD1 at MOI 100 did not significantly induce IL-6, transfection of bacterial RNA induced IL-6 in a dose-dependent manner. RNase A pre-treatment abolished the IL-6 response. Data for IL-8 are included in the **Supplementary Figure S9**. The RNA-mediated response is non-sequence specific since bacterial RNA from various staphylococci were able to induce the pro-inflammatory response in a similar manner.

To further verify our findings, we repeated identical experiments (co-culture with MOI 100 of viable bacteria and bacterial RNA transfection) with other clinical staphylococci isolates. For this purpose, we selected an invasive clinical *S. aureus* isolate from a skin and soft tissue infection (*S. aureus* SS-11291), an invasive clinical *Staphylococcus argenteus* SA147 [a member of the *S. aureus* complex, formerly classified as *S. aureus* ST2250 (37, 38)], a non-internalized clinical *S. aureus* (SA303) from a skin and soft tissue infection and a non-internalized clinical *Staphylococcus lugdunensis* HD1 (considered as coagulase-negative staphylococci) (39). In concordance to our initial findings, both invasive isolates, SS-11291 and SA147, induced IL-6 and IL-8 response at MOI 100, whereas the non-invasive isolates, SA303 and HD1, did not (**Figures 4A–C**). More importantly, transfection of bacterial RNA of the non-invasive SA303 and HD1 induced both IL-6 and IL-8, which was abolished upon RNase treatment prior to transfection (**Figure 4D** and **Supplementary Figure S7**).

### RNA in the Cytoplasm Is Responsible for the Pro-Inflammatory Response Following Bacterial Internalization

**Figure 5:**
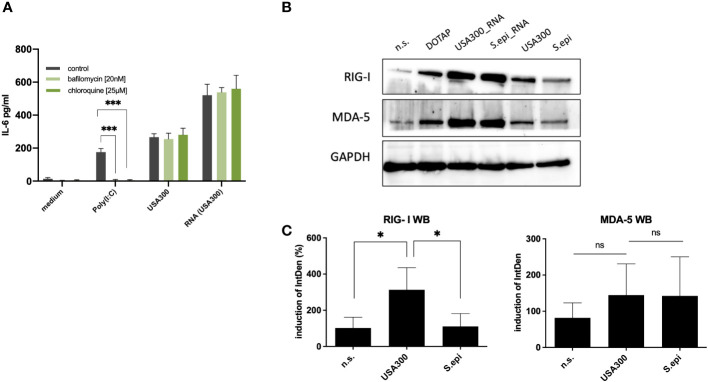
Cytoplasmic RNA sensors may be responsible for the induction of IL-6 by invasive staphylococci. **(A)** Inhibition with the endosomal TLR inhibitors bafilomycin and chloroquine did not affect the IL-6 response at MOI 100. **(B)** Both MDA-5 and RIG-I may be involved in the recognition of bacterial RNA following bacterial invasion, as quantified by western blot. Viable *S. aureus* USA300 induces more RIG-I and MDA5 in keratinocytes compared to viable *S. epidermidis* at MOI of 100. Transfection of *S. aureus* USA300 and *S. epidermidis* induced similar levels of RIG-I and MDA5. All experiments were performed as independent experiments in biological triplicates, each in technical duplicates, except for Western Blot (one representative experiment of three experiments). Raw file of the blot is provided in the supplementary. **(C)** Quantification of RIG-I and MDA-5 western blots of keratinocytes stimulated with viable *S. aureus* and *S. epidermidis* at MOI of 100. Quantification of protein bands were performed *via* ImageJ software. Data were analyzed by Graphpad Prism v9 (USA). The IntDen of protein band of interest was divided through IntDen of the respective houskeeping protein band. For calculation of induction mean of data of three unstimulated samples were set to 100 percentage. Statistically significant differences are indicated by * (*p < 0.5, ***p ≤ 0.001, ns, not significant).

Eukaryotic cells are equipped with both endoplasmic and cytoplasmic RNA sensors. In order to identify, which receptors may be involved in the recognition of bacterial RNA following bacterial internalization, we performed inhibition experiments using various PRR inhibitors. First, we inhibited the endosomal PRRs (TLR3, TLR7, TLR8 and TLR9) using bafilomycin and chloroquine (40), and Poly(I:C) (TLR3 ligand) as a positive control. The addition of 20nM bafilomycin and 25µM chloroquine were able to inhibit Poly(I:C) mediated induction of IL-6 and IL-8, but not the immune response towards viable bacteria of MOI 100 (**Figure 5A**). Therefore, it is unlikely that endosomal TLRs are largely involved in the sensing of internalized bacterial RNA in our experimental set-up.

The next step would be to inhibit the cytoplasmic RNA sensors, MDA5 and RIG-I. However, since specific inhibitors for RIG-I and MDA5 were not commercially available, we were not able to perform inhibition experiments for MDA5 and RIG-I receptors. As an alternative, we resort to protein expression level analysis using RT-qPCR and specific antibodies for MDA5 and RIG-I by Western Blot (**Figures 5B, C** and **Supplementary Figure S8**). Stimulation with viable *S. aureus* induced more RIG-I and MDA5 compared to stimulation with *S. epidermidis*, which was in line with the IL-6 and IL-8 response. Experimental attempt to inhibit the expression of MDA5 and RIG-I using siRNA was unsuccessful due to the cell toxicity effect at higher concentrations and non-significant inhibition at non-toxic concentrations.

## Discussion and Conclusion

The human skin is the outmost barrier of the human body and is exposed to environmental toxins, mechanical stress, pathogens and commensals. Besides providing a robust physical barrier, the skin is equipped to sense and respond to infection and tissue damage. As an immune response towards infections, the skin can initiate immune response to initiate pathogen clearance and to promote tissue regeneration (41). Keratinocytes are regarded as central players in the function as immune sentinels, which communicate and orchestrate T-cell mediated adaptive immune response. Excessive immune response in the skin can result in pathologic inflammatory response, disrupting tissue integrity and impairing the skin’s defense mechanisms. Therefore, a homeostatic state needs to be maintained to prevent overwhelming inflammatory response and keratinocytes must be able to distinguish between commensal and invading pathogens.

*S. aureus* and *S. epidermidis* are perfect model pathogens to study the mechanism by which keratinocytes differentiate between commensal and pathogen. Both *S. aureus* and *S. epidermidis* belong to the genus *Staphylococcus* and share a certain degree of similarity in the conserved microbial cellular structure. One important difference between *S. aureus* and *S. epidermidis* is their ability to invade cells and survive intracellularly. This suggests that intracellular sensing may play an important role in the sensing and differentiation of commensal and pathogenic staphylococci in keratinocytes. Indeed, our data strongly suggest that keratinocytes are activated in response to *S. aureus* invasion through an intracellular RNA-recognition mechanism.

The sensing of bacterial RNA has been attributed to the ability of the immune systems to detect and respond to microbial viability (42). To our knowledge, it is unclear whether keratinocytes can distinguish between viable or non-viable bacterial cells. In our experimental set-up, heat-inactivated bacteria were not able to induce significant IL-6 or IL-8 response, suggesting that bacterial viability is important for internalization/invasion, which in turn induces pro-inflammatory response in keratinocytes. The role of intracellular RNA receptors in differentiating between viable and dead pathogens has been demonstrated for other bacteria and fungi (43–45). Innate immune cells can detect bacterial mRNA, common to all bacteria, to initiate an immune response (46, 47). These bacterial mRNA are considered as the signature of microbial viability and are often classified as a special class of pathogen-associated molecular pattern (PAMP), called vita-PAMPs (47). Although the role of vita-PAMPs has been studied for other Gram-positive bacteria, such as *Streptococcus pyogenes* (42, 48), the role of bacterial mRNA sensing for *S. aureus* has not been explored.

In our experiments, we observed differences in the secreted IL-6 and IL-8 levels, which correlated with the internalization of staphylococci. Although the exact roles of IL-6 and IL-8 in terms of cutaneous defense are not yet fully elucidated, IL-6 and IL-8 have been found to contribute to local wound healing for their ability to promote migration and proliferation of keratinocytes (49, 50). Moreover, IL-6 might prevent *S. aureus* from spreading to yet unaffected healthy host cells by promoting keratinocyte differentiation and thus, accelerate the disposal of already infected and surrounding tissue (51, 52). During differentiation, infected cells undergo cell death, migrate to the upper layers of the epidermis and eventually, form a cornified barrier (53), which is beneficial considering that *S. aureus* usually requires lesion in the epithelial barrier as a port of entry for infection (54). In contrast to other findings, although the cell line used in our experiments express TLR2, we did not see a strong induction of TLR2-mediated response through secreted mediators, which have been demonstrated in experiments using primary keratinocytes by other groups (1). Although we do not have any definitive explanation for this discrepancy, differences in the TLR2 expression due to differentiation state of the cells or culture conditions (removal of cellular debris by centrifugation and sterile filtering of the supernatant) may have an influence on the expression of PRR in keratinocytes.

The role of IL-6 in the pathophysiology of the skin has been extensively investigated. IL-6 deficiency has been attributed to exacerbation of skin inflammation in an *in vivo* murine model (55). Furthermore, lower levels of IL-6 in the skin have been linked to exacerbation of atopic dermatitis, a skin condition associated with a propensity for *S. aureus* colonization and infections. On the other hand, patients with psoriasis are less susceptible to *S. aureus* infections, and elevated IL-6 levels have been associated with this skin condition and may be a potential target for psoriasis therapy (56–59). IL-6 also performs an important function in the immunological function of the skin by linking the innate to the acquired immune response (60, 61).

IL-8 is involved in recruitments of immune cells to initiate and process further inflammatory response cascade. Indeed, many immune cells, such as neutrophils, T lymphocytes, mast cells, dermal macrophages, endothelial cells and keratinocytes possess IL-8 binding sites (62). The chemotactic properties have been attributed to certain processes, such as cell migration and wound healing (50). Numerous studies have shown that infection of keratinocytes with *S. aureus* induce IL-8 (63, 64). In a murine model, IL-8 has been demonstrated to be important in neutrophil recruitment, which is essential for bacterial clearing in the context of *S. aureus* colonization/infection (65).Our findings may be relevant to understand the host-bacterial interaction, which may play a role in establishing *S. aureus* colonization. Our previous studies demonstrated that *S. aureus* persistent carriers expressed less antimicrobial peptides than non-carriers, specifically hBD-3, and that this phenotype was linked with genetic polymorphisms in the *DEFB1* gene (8, 9). In addition, the Th1/Th17-mediated immune response in whole blood was less profound in *S. aureus* persistent carriers than in non-carriers following *ex vivo* stimulation with viable *S. aureus* (11). However, it is unclear how the immune system of the skin differentiates between commensal and pathogenic bacteria. In this study, we could demonstrate that only *S. aureus* induces pro-inflammatory response in keratinocytes. Together with our previous finding, this study adds to the body of evidence that immunological processes and bacterial-host interaction play an important role in the pathophysiology of *S. aureus* colonization.

Our study has limitations; the study was conducted in an *in vitro* setting, so that *in vivo* validation is still needed. The experiments were conducted using an immortalized keratinocyte cell line. We chose this experimental set-up (using a characterized cell line) to ensure comparability between results as the genetic background of keratinocyte donors may influence the immunological function (9). In our experiments, we did not see a strong induction of TLR2-mediated immune response by *S. aureus* USA300, although HaCaT keratinocyte has been shown to express and react to TLR2 (1). The expression of TLR2 may be lower in this cell lines than primary keratinocytes and validation studies with other cell types and primary cells are necessary to verify our findings. In our experimental set-up, only several bacterial isolates were used, and species heterogeneity was not investigated. Therefore, our data should be interpreted carefully and further validatory experiments are necessary to verify the generalizability of our findings. Nonetheless, our study investigated the host-bacteria interaction using viable bacteria instead of inactivated bacterial cells. As demonstrated, heat-killed bacteria may not be suitable to study host-pathogen interaction since dead bacteria may not express specific proteins, which may be relevant in the overall immunological context.

In the past, keratinocytes were mainly considered as a physical barrier rather than active participants of the immune system. Our data imply that keratinocytes respond differentially to invasive and non-invasive pathogens of the skin. This effect is not specific for a single pathogen but rather non-specific and is mediated *via* “invasion” of bacterial RNA into the intracellular compartment. Furthermore, this study demonstrated that non-invasive staphylococci do not induce a profound pro-inflammatory reaction, which may be necessary to promote bacterial elimination and allow these bacteria to colonize the skin as commensals.

## Data Availability Statement

The original contributions presented in the study are included in the article/**Supplementary Material**. Further inquiries can be directed to the corresponding author.

## Author Contributions

QN, AS, KH, and DN conceived and planned the experiments. QN, LF, and AS performed the experiments. QN, AS, and DN analyzed the experiments. KH and TE contributed to the interpretation of the results. QN, DH, and DN drafted and finalized the manuscript. All authors provided critical feedback and contributed to the final manuscript. All authors approved the final submitted version of the manuscript.

## Funding

This study was supported by a grant number: 80295MDQUN/TI07.003_Ngo (MD stipend) from the German Center for Infection Research (DZIF) for QN with KH and DN as supervisor for this funded research project.

## Conflict of Interest

The authors declare that the research was conducted in the absence of any commercial or financial relationships that could be construed as a potential conflict of interest.

## Publisher’s Note

All claims expressed in this article are solely those of the authors and do not necessarily represent those of their affiliated organizations, or those of the publisher, the editors and the reviewers. Any product that may be evaluated in this article, or claim that may be made by its manufacturer, is not guaranteed or endorsed by the publisher.
