# Corrigendum: Inflammatory response against *Staphylococcus aureus* via intracellular sensing of nucleic acids in keratinocytes

**DOI:** 10.3389/fimmu.2024.1330357

**Published:** 2024-03-05

**Authors:** Quang Vinh Ngo, Larissa Faass, Aline Sähr, Dagmar Hildebrand, Tatjana Eigenbrod, Klaus Heeg, Dennis Nurjadi

**Affiliations:** ^1^Department of Infectious Diseases, Medical Microbiology and Hygiene, Heidelberg University Hospital, Heidelberg, Germany; ^2^Deutsches Zentrum für Infektionsforschung (DZIF), Department of Infectious Diseases, Heidelberg University Hospital, Heidelberg, Germany; ^3^Max von Pettenkofer Institute, Chair for Medical Microbiology and Hygiene, Ludwig Maximilians University Munich, Munich, Germany

**Keywords:** *Staphylococcus aureus*, *Staphylococcus epidermidis*, keratinocyte, skin immune response, bacterial RNA, host-pathogen interaction

In the published article, there was an error in the published **Figure 5**. In the original published version of **Figure 5**, we separated the Western blot (panel B) of the whole bacterial cells and the transfected bacterial RNA for illustrative purposes. As a result, the negative control was duplicated as the Western blot was run as a single blot for ease of comparison/illustration. However, this was not clearly stated in the caption or in the figure, which may have given the impression that the blot could have been ‘manipulated’. To avoid further misunderstanding, we have decided to replace the Western blot figure in panel B with an untrimmed and identical version of the same blot. For transparency, we have included the original blots and other raw data as additional supplementary files. The corrected **Figure 5** and its caption appear below.

**Figure 5:**
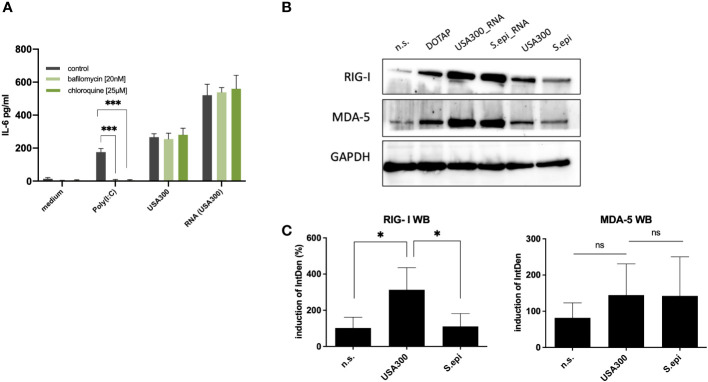
Cytoplasmic RNA sensors may be responsible for the induction of IL-6 by invasive staphylococci. **(A)** Inhibition with the endosomal TLR inhibitors bafilomycin and chloroquine did not affect the IL-6 response at MOI 100. **(B)** Both MDA-5 and RIG-I may be involved in the recognition of bacterial RNA following bacterial invasion, as quantified by western blot. Viable *S. aureus* USA300 induces more RIG-I and MDA5 in keratinocytes compared to viable *S. epidermidis* at MOI of 100. Transfection of *S. aureus* USA300 and *S. epidermidis* induced similar levels of RIG-I and MDA5. All experiments were performed as independent experiments in biological triplicates, each in technical duplicates, except for Western Blot (one representative experiment of three experiments). Raw file of the blot is provided in the supplementary. **(C)** Quantification of RIG-I and MDA-5 western blots of keratinocytes stimulated with viable *S. aureus* and *S. epidermidis* at MOI of 100. Quantification of protein bands were performed *via* ImageJ software. Data were analyzed by Graphpad Prism v9 (USA). The IntDen of protein band of interest was divided through IntDen of the respective houskeeping protein band. For calculation of induction mean of data of three unstimulated samples were set to 100 percentage. Statistically significant differences are indicated by * (*p < 0.5, ***p ≤ 0.001, ns, not significant).

The authors apologize for this error and state that this does not change the scientific conclusions of the article in any way. The original article has been updated.

