# Supplementary material for: Inflammatory Response Against Staphylococcus aureus via Intracellular Sensing of Nucleic Acids in Keratinocytes
Source: Front Immunol. 2022 Feb 24;13:828626. doi: 10.3389/fimmu.2022.828626 (PMC8907419; doi:10.3389/fimmu.2022.828626)
Supplement: Supplementary file 1 [file DataSheet_1.pdf]

Supplementary Material

Inflammatory response against *Staphylococcus aureus* via intracellular sensing of nucleic acids in keratinocytes

Quang Vinh Ngo[1,2], Larissa Faass[1,3], Aline Sähr[1], Dagmar Hildebrand[1], Tatjana Eigenbrod[1], Klaus Heeg[1,2], Dennis Nurjadi[1,2]

[1] Department of Infectious Diseases, Medical Microbiology and Hygiene, Heidelberg University Hospital, Heidelberg, Germany

[2] Deutsches Zentrum für Infektionsforschung (DZIF), Department of Infectious Diseases, Heidelberg University Hospital, Heidelberg, Germany

[3] Max von Pettenkofer Institute, Chair for Medical Microbiology and Hygiene, Ludwig Maximilians University Munich, Munich, Germany.

Content

Supplementary Table S1. Bacterial isolates used in this study. 1

Supplementary Table S2. Primers used in the study. 2

Supplementary Figure S1. Quantification of internalized bacterial cells by flow cytometry. Keratinocyte from a confluent monolayer was trypsinized and visualized by flow cytometry. 3

Supplementary Figure S2. Toxicity of Cytochalasin-D by MTT assay. 4

Supplementary Figure S3. Expression of antimicrobial peptides in immortalized keratinocytes (HaCaT) stimulated with vital *Staphylococcus aureus* and *Staphylococcus epidermidis*. 5

Supplementary Figure S4. Cell viability after bacterial stimulation with *Staphylococcus aureus* USA300 and *Staphylococcus epidermidis*. 6

Supplementary Figure S5. Quantification of internalized bacteria by manual colony counting. 7

Supplementary Figure S6. Cell survival assay by MTT-test. 8

Supplementary Figure S7. IL-8 response in immortalized keratinocytes following infection with *Staphylococcus aureus* and *Staphylococcus epidermidis*. 9

Supplementary Figure S8. IL-8 response in immortalized keratinocytes following infection with various staphylococci. Similar to *Staphylococcus aureus* USA300, both invasive isolates (*Staphylococcus argenteus* SA147 and *S. aureus* SS-11921) 10

Supplementary Figure S9. RIG-I and MDA-5 mRNA expression in keratinocytes following internalization of staphylococcal RNA. 11

References 12

Supplementary Table S1. Bacterial isolates used in this study.

**Isolate-ID (This study)**	**Origin**	**Coagulase**	**Invasive**	**Reference**
Staphylococcus aureus USA300 FPR3757 (BAA-1556)	type strain; ST8, t008, Panton-Valentine Leukocidin positive MRSA	coagulase positive	yes	(1)
Staphylococcus epidermidis ATCC®35894	type strain	coagulase negative	no	n/a
Staphylococcus argenteus SA147 (S. aureus complex)	*S. argenteus* clinical isolate (skin and soft tissue infection), ST2250, a member of the *Staphylococcus aureus* complex	coagulase positive	yes	this study
Staphylococcus aureus SA303	*S. aureus* clinical isolate (skin and soft tissue infection), non-invasive, ST182	coagulase positive	no	this study
Staphylococcus aureus DU5883*	laboratory strain, fibronectin deficient 8325-4	coagulase positive	no	(2)
Staphylococcus aureus 8325-4*	laboratory strain	coagulase positive	yes	(2)
Staphylococcus aureus SS-11921	*S. aureus* clinical isolate (wound infection)	coagulase positive	yes	this study
Staphylococcus lugdunensis HD1	*S. lugdunensis* clinnical isolate (wound infection)	coagulase negative	no	this study
Staphylococcus carnosus TM300	type strain, negative control for invasivity assay (non-invasive coagulase negative *Staphylococcus* sp.)	coagulase negative	no	(3)
S. aureus Cowan I (ATCC 12598)	type strain, control for invasivity assay (invasive *S. aureus*)	coagulase positive	yes	(4)

*Isolate *S. aureus* 8325-4 and DU5883 were kindly provided by Frieder Schaumburg, Medical Microbiology, University Hospital Münster, Germany.

Supplementary Table S2. Primers used in the study.

Target gene	Sequence 5'-3'
ß-Actin fw	aga gct acg agc tgc ctg ac
ß-Actin rv	agc act gtg ttg gcg tac ag
hBD-1 fw	tga gtg ttg cct gcc agt
hBD-1 rv	tct tct ggt cac tcc cag
hBD-2 fw	tga tgc ctc ttc cag gtg tt
hBD-2 rv	tga tga ggg agc cct ttc tg
hBD-3 fw	ttg ctc ttc ctg ttt ttg gtg
hBD-3 rv	cgc ctc tga ctc tgc aat aa
MDA5 fw	tcg aat ggg tat tcc aca gac g
MDA5 rv	gtg gcg act gtc ctc tga a
RIG-I fw	tgt gct cct aca ggt tgt gga
RIG-I rv	cac tgg gat ctg att cgc aaa a

Abbrv. hBD=human beta defensin

Supplementary Figure S1. Quantification of internalized bacterial cells by flow cytometry. Keratinocyte from a confluent monolayer was trypsinized and visualized by flow cytometry.

A Gated keratinocyte population in the forward (FSC) and side scatter (SSC). **B** After co-culture with CFSE-labelled bacteria for 2 hours, gentamicin and lysostaphin was added and washed multiple times with PBS to remove extracellular bacteria. The invasion property of several staphylococci were assessed by quantification of CFSE positive subpopulation from the total gated population (**A**). Both *Staphylococcus epidermidis* and *Staphylococcus aureus* DU5883 did not show significant internalization at MOI of 10 and 100, while *S. aureus* USA300 and 8325-4 infected 40.2% and 35.3% of the gated population at MOI 100, respectively. Figures displayed are from one representative experiment.

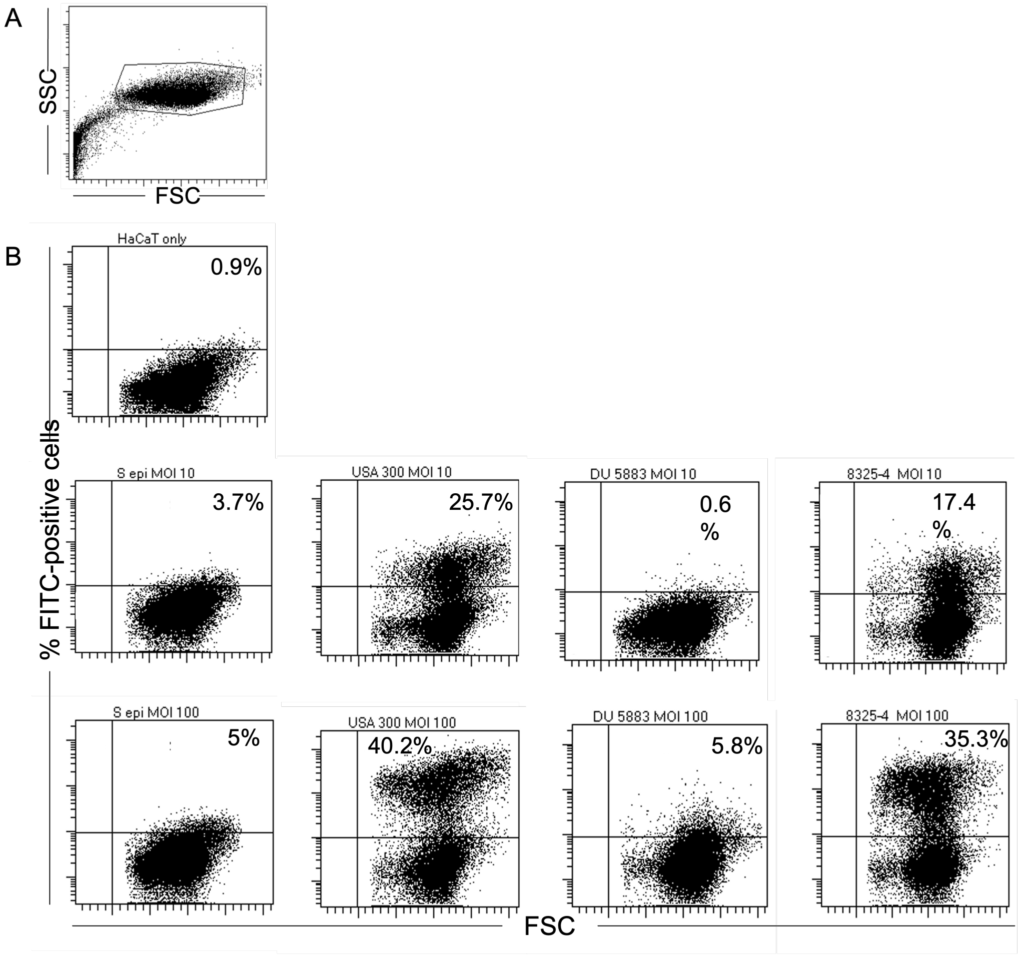

Supplementary Figure S2. Toxicity of Cytochalasin-D by MTT assay.

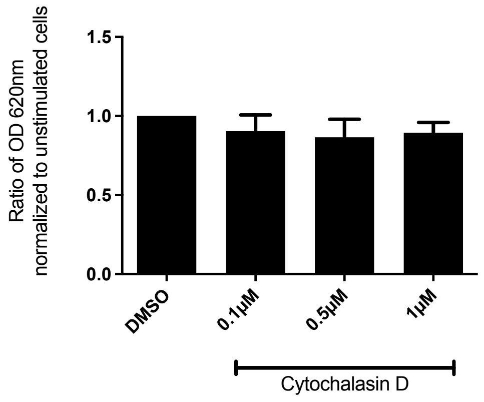

Supplementary Figure S3. Expression of antimicrobial peptides in immortalized keratinocytes (HaCaT) stimulated with vital *Staphylococcus aureus* and *Staphylococcus epidermidis*.

Data shown as collective data from three independent experiments performed as duplicates

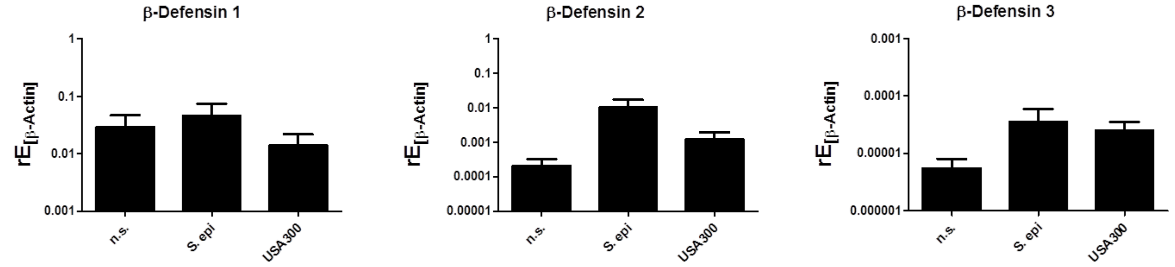

Supplementary Figure S4. Cell viability after bacterial stimulation with *Staphylococcus aureus* USA300 and *Staphylococcus epidermidis*.

Cell viability was analyzed using flow cytometry (Annexin V and Propidium Iodide (PI) staining), expressed as relative % to the gated population (in the forward and side scatter plot). There were no significant differences in cell viability after stimulation with viable *S. aureus* USA300 and *S. epidermidis*.

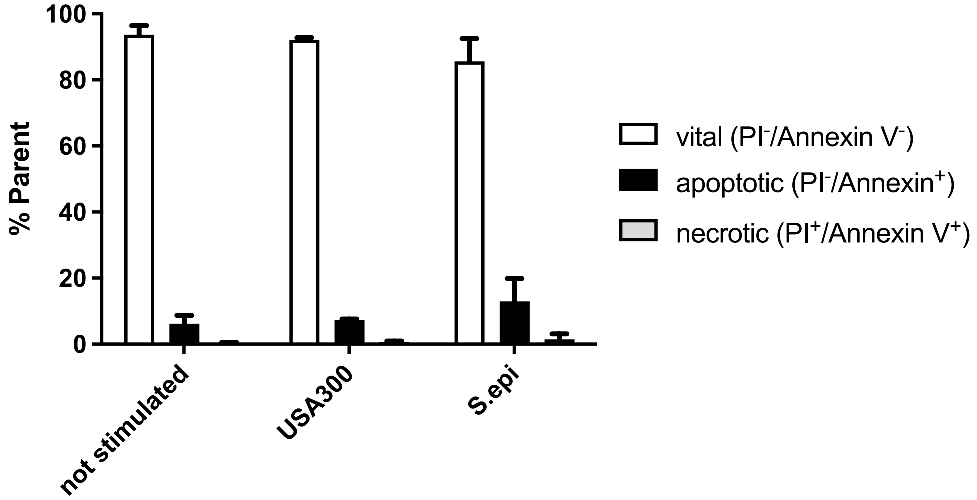

Supplementary Figure S5. Quantification of internalized bacteria by manual colony counting.

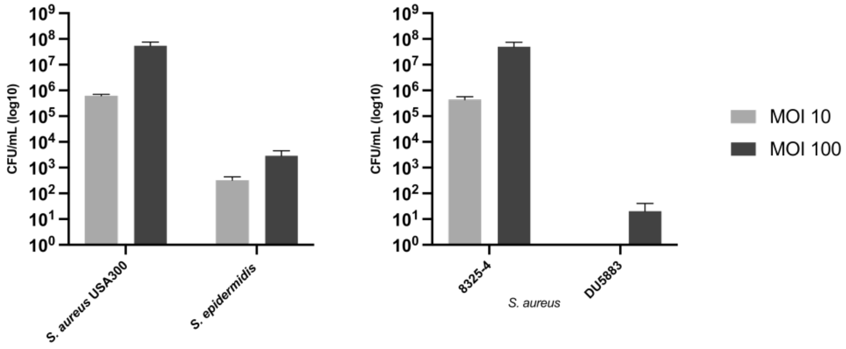

Supplementary Figure S6. Cell survival assay by MTT-test.

The effect of various concentration of bacteria conditioned medium (BCM) based on DMEM supplemented with 10% FCS without antibiotics expressed as percentage compared to the unstimulated control (100%). Incubation with up to 50% BCM for 24h at 37°C with 5% CO_2 did not significantly reduce cell metabolic properties (i.e. no toxic effect). Data shown is a representative data from one experiment performed as technical duplicate.

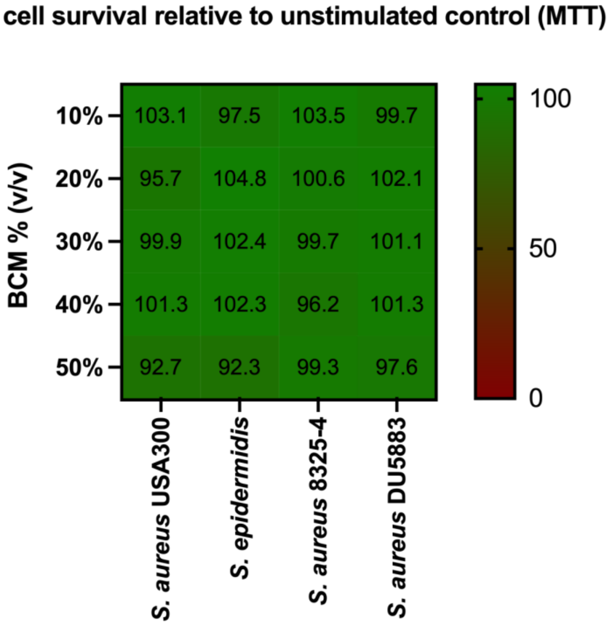

Supplementary Figure S7. IL-8 response in immortalized keratinocytes following infection with *Staphylococcus aureus* and *Staphylococcus epidermidis*. A. Co-culture of HaCaT with *S. epidermidis* of MOI 100 did not induce IL-8 response, whereas stimulation with transfected bacterial RNA induced IL-8 response in a dose-dependent manner similar to the IL-6 response (comparison Figure 3). Heat inactivation and RNase treatment abolished the IL-8 response. **B.** Co-culture of HaCaT with *S. aureus* of MOI 100 induce IL-8 response, similar to the IL-6 response (Figure 3, main text). Transfection of RNA induces IL-8 response in a dose-dependent manner, which was abolished when RNA was treated with RNase or heat-inactivated prior to transfection.

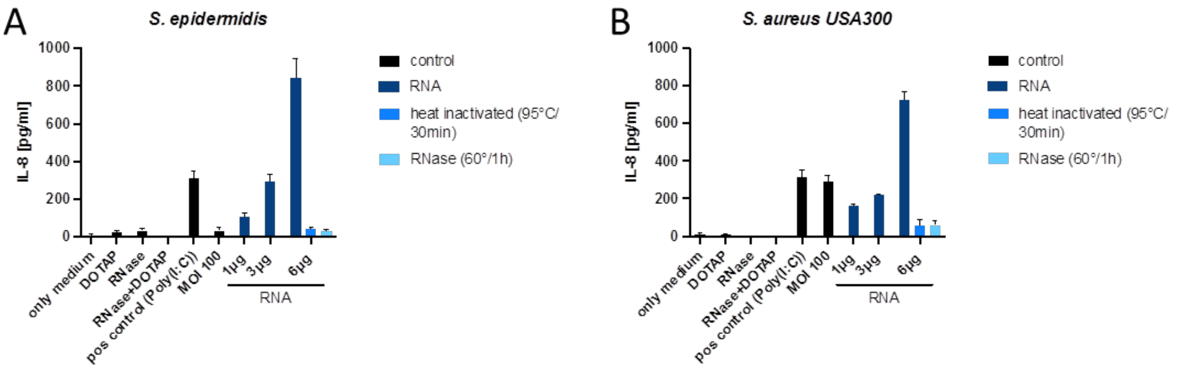

Supplementary Figure S8. IL-8 response in immortalized keratinocytes following infection with various staphylococci. Similar to *Staphylococcus aureus* USA300, both invasive isolates (*Staphylococcus argenteus* SA147 and *S. aureus* SS-11921) induced IL-8 response following co-culture with vital bacteria at MOI 100, which was abolished by RNase treatment and heat inactivation (top panels). The non-invasive *S. aureus* SA303 and *Staphylococcus lugdunensis* HD1 did not induce significant IL-8 response at MOI 100 (vital bacteria) but transfection of the RNA induce IL-8 in a dose-dependent manner.

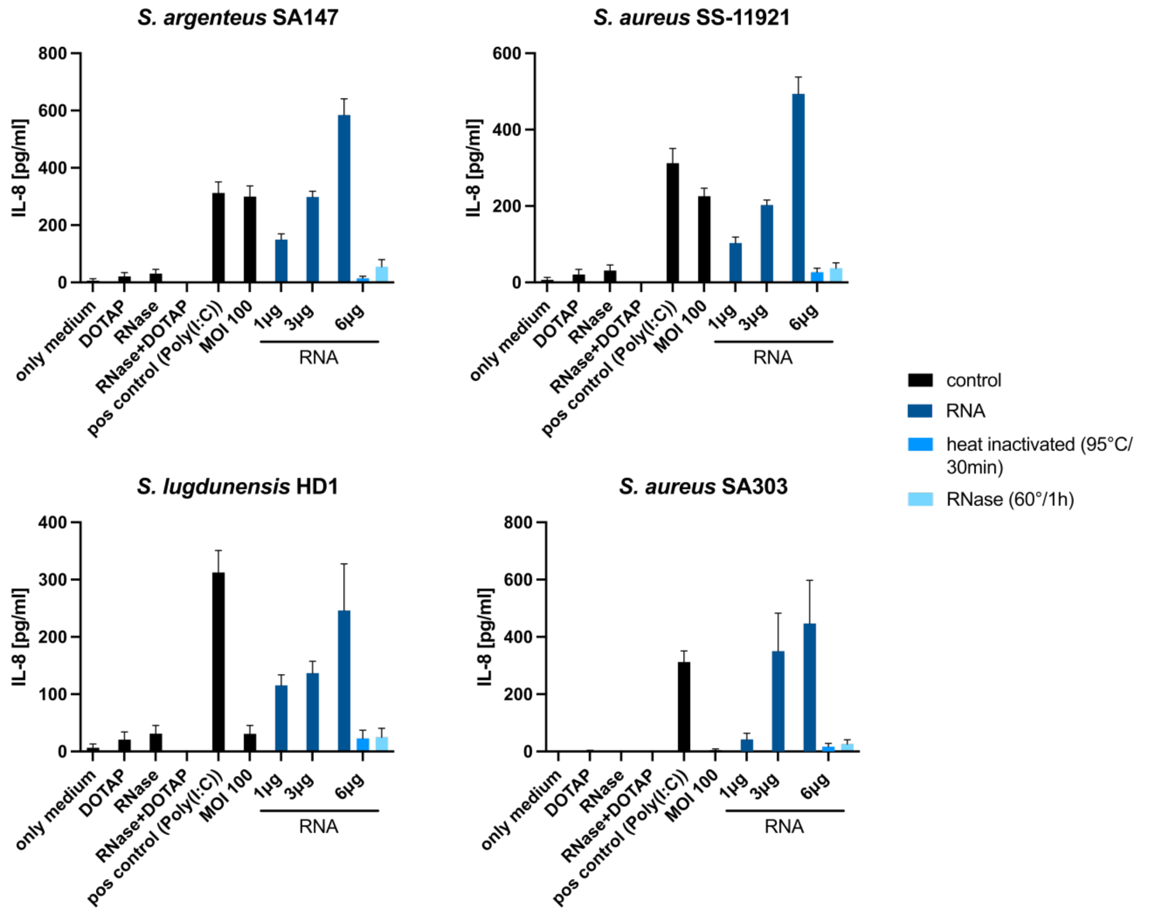

Supplementary Figure S9. RIG-I and MDA5 mRNA expression in keratinocytes following internalization of staphylococcal RNA.

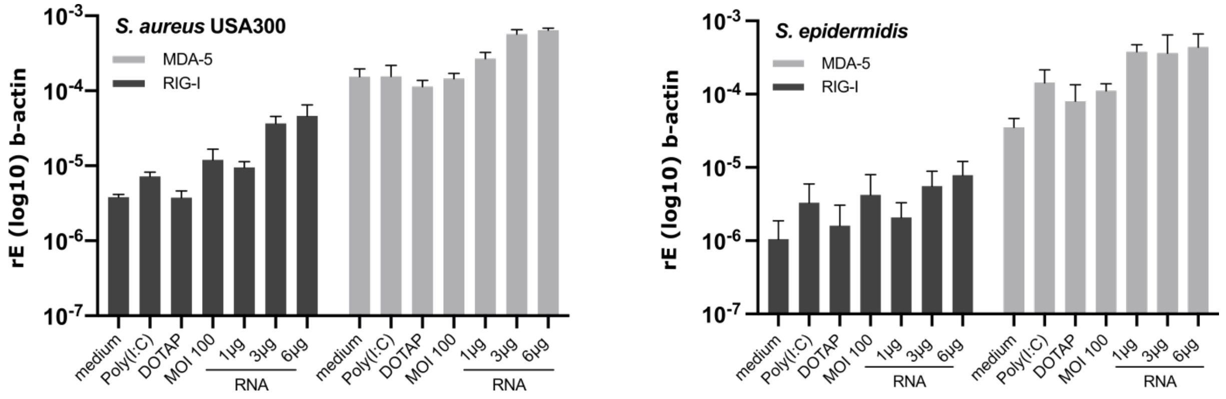

References

1. Diep BA, Gill SR, Chang RF, Phan TH, Chen JH, Davidson MG, et al. Complete genome sequence of USA300, an epidemic clone of community-acquired meticillin-resistant Staphylococcus aureus. Lancet. 2006;367(9512):731-9.

2. Dziewanowska K, Patti JM, Deobald CF, Bayles KW, Trumble WR, Bohach GA. Fibronectin binding protein and host cell tyrosine kinase are required for internalization of Staphylococcus aureus by epithelial cells. Infect Immun. 1999;67(9):4673-8.

3. Wagner E, Doskar J, Gotz F. Physical and genetic map of the genome of Staphylococcus carnosus TM300. Microbiology (Reading). 1998;144 (Pt 2):509-17.

4. Levinson AI, Tar L, Carafa C, Haidar M. Staphylococcus aureus Cowan I. Potent stimulus of immunoglobulin M rheumatoid factor production. J Clin Invest. 1986;78(3):612-7.
